# Supplementary material for: Dynamic magnetic crossover at the origin of the hidden-order in van der Waals antiferromagnet CrSBr
Source: Nat Commun. 2022 Aug 12;13:4745. doi: 10.1038/s41467-022-32290-4 (PMC9374657; doi:10.1038/s41467-022-32290-4)
Supplement: Supplementary file 1 — Supplementary Information [file 41467_2022_32290_MOESM1_ESM.pdf]

Supplementary Information: Dynamic Magnetic Crossover at the Origin of the Hidden-Order in van der Waals Antiferromagnet CrSBr

Sara A. López-Paz[1,2,*], Zurab Guguchia[3], Vladimir Y. Pomjakushin[4], Catherine Witteveen[1,2], Antonio Cervellino[5], Hubertus Luetkens[3], Nicola Casati[4], Alberto F. Morpurgo[2,6], and Fabian O. von Rohr[2,*]

[1]Department of Chemistry, University of Zurich, CH-8057, Switzerland
[2]Department of Quantum Matter Physics, University of Geneva, CH-1211 Geneva, Switzerland
[3]Laboratory for Muon Spin Spectroscopy, Paul Scherrer Institute, CH-5232 Villigen PSI, Switzerland [4]Laboratory for Neutron Scattering and Imaging, Paul Scherrer Institute, CH-5232 Villigen PSI, Switzerland [5]Laboratory for Synchrotron Radiation - Condensed Matter, Paul Scherrer Institut, CH-5232 Villigen, Switzerland [6]Department of Applied Physics, University of Geneva, CH-1211 Geneva, Switzerland
[*]sara.lopezpaz@unige.ch and fabian.vonrohr@unige.ch

Supplementary Note 1: Neutron powder diffraction experiments

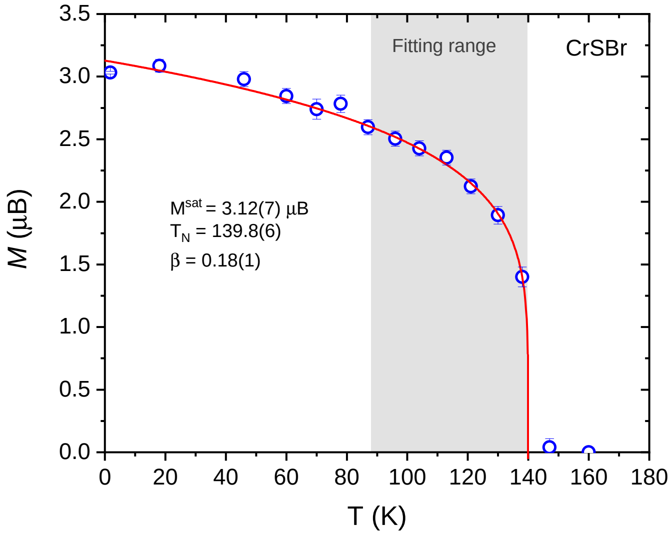

Supplementary Figure 1. Temperature dependence of the refined magnetic moment from the neutron diffraction data (blue cirles). The fitting to a power law $M \propto (T_c - T)^{\beta}$ is shown as a red continuous line, extrapolated to base temperature. The temperature region considered in the fitting is shown as a grey dashed area.

		T = 160 K	T = 1.8 K
	a (Å)	3.5072(1)	3.5127(1)
	b (Å)	4.7492(2)	4.7458(1)
	c (Å)	7.9362(2)	7.9131(2)
	V (Å^3)	132.19(1)	131.92(1)
Atom	Site		
Cr	2b (0.25, 0.75, z)		
	z	-0.1251(7)	-0.1269(6)
	B_{iso} (Å^3)	0.36(7)	0.22(6)
S	2a (0.75, 0.75, z)		
	z	0.0764(9)	0.0739(9)
	B_{iso}	0.2(1)	0.21(9)
Br	2a (0.75, 0.75, z)		
	z	-0.3511(6)	-0.3535(6)
	B_{iso}	0.66(5)	0.40(3)
	R_p	4.02	4.38
	R_{wp}	5.14	5.59
	R_{Bragg}	3.90	3.48
	χ^2	1.44	1.71

Supplementary Table 1. Cell and structural parameters obtained from Rietveld refinements of the NPD data (λ = 2.449 Å), at T = 160 K and T = 1.8 K for CrSBr, together with the agreement factors.

$Symm.$	x,y,z	$-x,y+1/2,-z$
BASR	0 1 0	0 1 0
BASI	0 0 0	0 0 0

Supplementary Table 2. Irreducible representation used for the refinement of the magnetic structure considering a single magnetic site at the Cr 2b position (0.25, 0.75, z).

Supplementary Note 2: Magnetization measurements

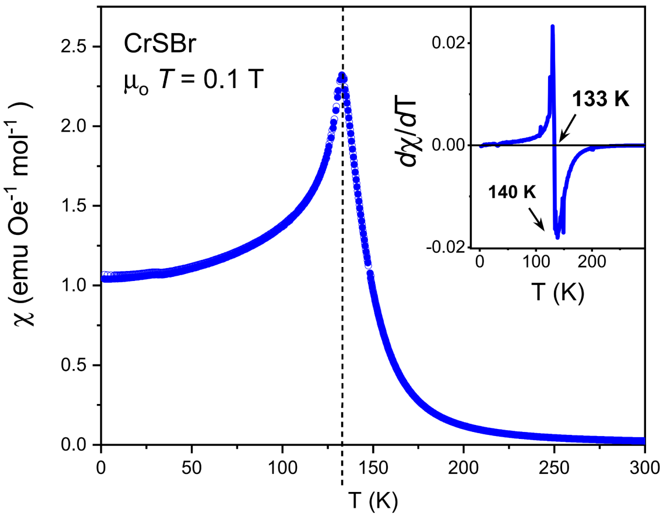

Supplementary Figure 2. Magnetic susceptibility for powder CrSBr under a 0.1 T magnetic field in zero field cooling (ZFC, filled circles) and field cooling (FC, empty circles) modes. Inset show the derivative of the ZFC curve.

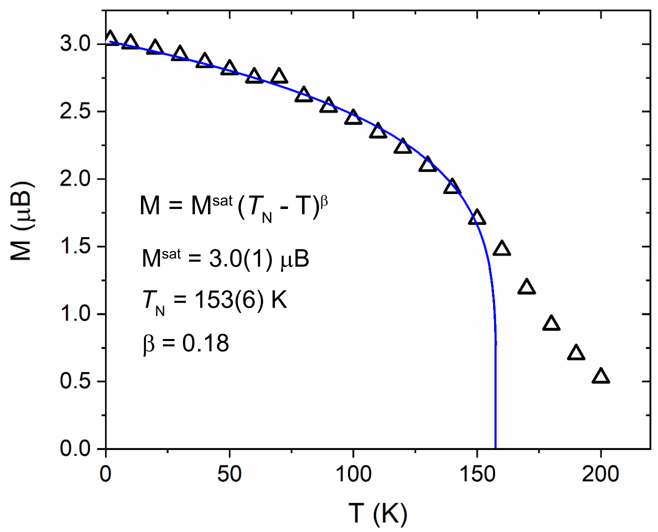

Supplementary Figure 3. Temperature dependence of the saturation magnetization M(3T) –open triangles- and fitting to a power law $M \propto (T_c - T)^{\beta}$ with a fixed critical exponent of $\beta = 0.18$ as derived from the neutron diffraction data in the 1.8 K-200 K temperature range.

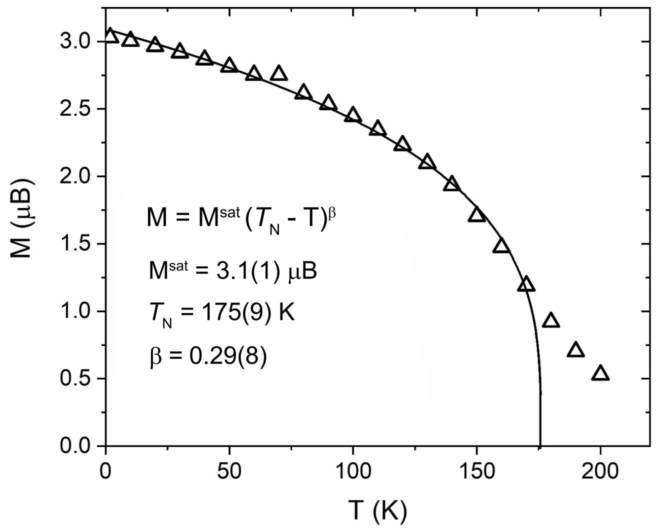

Supplementary Figure 4. Temperature dependence of the saturation magnetization M(3T) –open triangles- and free fitting to a power law in the 1.8 K- 200 K temperature range.

Supplementary Note 3: Synchrotron X-ray diffraction measurements

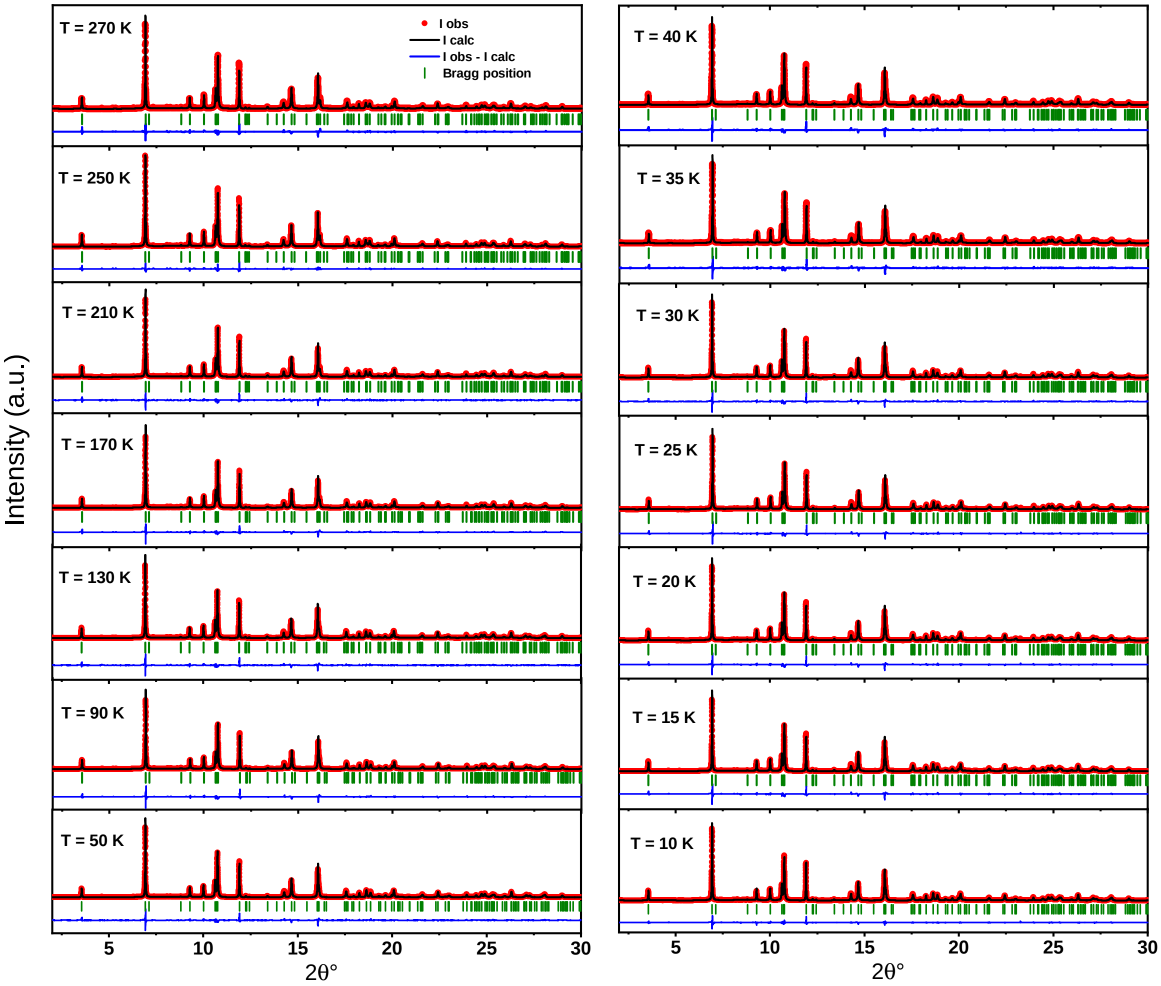

Supplementary Figure 5. Rietveld refinement of the SXRD data (λ = 0.492 Å) at different temperatures for CrSBr within the *Pmnm* space group.

T(K)	a (Å)	b (Å)	c (Å)	V (Å^3)	R_p	R_{wp}	R_{Bragg}
270	3.50440(4)	4.75927(5)	7.9546(1)	132.67(1)	5.30	7.59	5.21
250	3.50439(4)	4.75787(5)	7.9511(1)	132.57(1)	5.22	7.43	4.75
210	3.50521(4)	4.75502(5)	7.9448(1)	132.41(1)	5.18	7.32	4.62
170	3.50686(4)	4.75160(5)	7.9396(5)	132.30(1)	6.00	7.91	5.23
130	3.50901(4)	4.74839(4)	7.9343(1)	132.20(1)	5.78	7.84	4.42
90	3.50974(4)	4.74603(5)	7.9260(1)	132.02(1)	8.75	12.2	8.01
50	3.51036(4)	4.74594(5)	7.9200(1)	131.94(1)	5.65	7.70	4.21
40	3.51030(4)	4.74584(5)	7.9187(1)	131.92(1)	5.83	7.87	4.48
36	3.51031(4)	4.74584(5)	7.9182(1)	131.91(1)	5.89	7.90	4.45
30	3.51029(4)	4.74583(5)	7.9175(1)	131.90(1)	6.18	8.15	5.03
25	3.51028(4)	4.74581(5)	7.9172(1)	131.89(1)	6.14	8.13	4.76
20	3.51026(4)	4.74581(5)	7.9168(5)	131.88(1)	6.11	8.12	5.02
15	3.51025(4)	4.74581(5)	7.9165(1)	131.88(1)	6.05	8.01	4.86
10	3.51023(5)	4.74575(6)	7.9164(1)	131.87(1)	6.65	8.57	6.95

Supplementary Table 3. Cell parameters and cell volume obtained from Rietveld refinement of the SXRD data for CrSBr at different temperatures, together with the agreement factors.

Supplementary Note 4: μSR experiment and analysis

Zero field and weak transverse field (30 G) μSR spectra were acquired at the GPS (πM3 beamline) instrument at the Paul Scherrer Institute, allowing essentially a background free μSR measurement at ambient conditions. The sample was mounted in a He gas-flow cryostat and the temperature was varied between 5 and 200 K.

Analysis of weak TF-µSR data.

The weak TF asymmetry spectra were analyzed using the following function:

$$A^{TF}(t) = A_o \cdot exp(-\lambda t) \cdot cos(\omega t + \phi)$$

Eq.1

Where t is the time after muon implantation, A(t) is the time-dependent asymmetry, A_0 is the amplitude of the oscillating component and λ is the exponential damping rate due to paramagnetic spin fluctuations and/or nuclear dipolar moments. $\omega = 2\pi\nu\mu$ is the Larmor precession frequency set by the strength of the transverse magnetic field, and ϕ is a phase offset. The magnetically ordered volume fraction at each temperature was calculated from the refined amplitude $A(T)_0$ as

$$V_M = 1 - A_0(T)/A_0(T_{max})$$

Eq.2

Where $A_0(T_{max})$ is the amplitude in the paramagnetic phase at high temperature.

Analysis of ZF-μSR data.

The ZF spectra in the paramagnetic state (130 K < T < 200 K) were fitted using a single exponential function of the form:

$$A^{ZF}(t) = A_0 \cdot exp(-\lambda_{pm} t)$$

Eq.3

The ZF spectra in the 5 K < T < 130 K temperature range were fitted using the following function:

$$A^{ZF}(t) = A_0 \cdot f_{osc} \cdot cos(\gamma_\mu B_\mu t + \phi) \cdot exp(-\lambda_1 t) + A_0 \cdot f_L \cdot exp(-\lambda_2 t)$$

Eq.4

The first and second terms describe the oscillating component (f_{osc}) and the slowly relaxing longitudinal component (f_L), respectively. The longitudinal component arises due to muons experiencing local field components which are parallel to the initial muon spin polarization. λ_1 and λ_2 are the oscillating and non-oscillating relaxation rates, respectively. B_μ is the internal magnetic field at the muon site and γ_μ = 135.5 MHz/T is the muon gyromagnetic ratio.

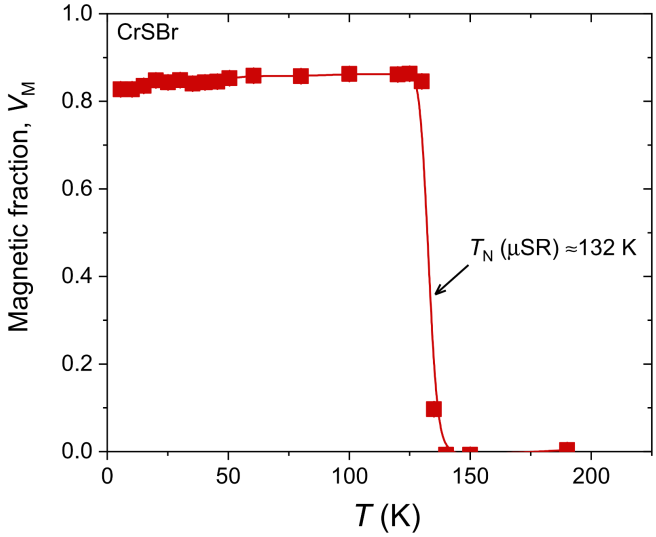

Supplementary Figure 6. Magnetic volume fraction as a function of temperature for CrSBr as derived from the weak TF-μSR measurements.

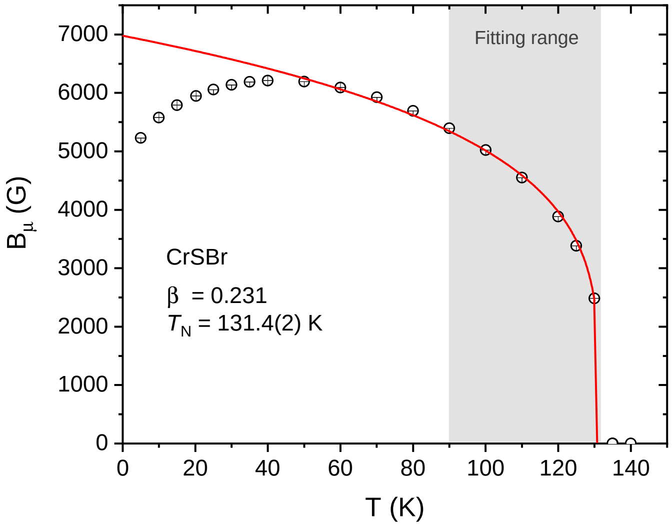

Supplementary Figure 7. Temperature dependence of the internal magnetic field as derived from the ZF-μSR data (black cirles). The fitting to a power law $M \propto (T_c - T)^\beta$ using a fixed exponent of $\beta = 0.231$ according to the 2DXY model is shown as a red continuous line, extrapolated to base temperature. The temperature region considered in the fitting is shown as a grey dashed area.
